# Simulating the mechanisms of serrated flow in interstitial alloys with atomic resolution over diffusive timescales

**DOI:** 10.1038/s41467-020-15085-3

**Published:** 2020-03-06

**Authors:** Yue Zhao, Lucile Dezerald, Marta Pozuelo, Xinran Zhou, Jaime Marian

**Affiliations:** 1 Department of Materials Science and Engineering, University of California Los Angeles, Los Angeles, CA 90095 USA; 2 Department of Materials Science and Engineering, Institut Jean Lamour, Université de Lorraine, F-54011 Nancy, France; 3 Department of Mechanical and Aerospace Engineering University of California Los Angeles, Los Angeles, CA 90095 USA

**Keywords:** Metals and alloys, Computational methods

## Abstract

The Portevin-Le Chatelier (PLC) effect is a phenomenon by which plastic slip in metallic materials becomes unstable, resulting in jerky flow and the onset of inhomogeneous deformation. The PLC effect is thought to be fundamentally caused by the dynamic interplay between dislocations and solute atoms. However, this interplay is almost always inaccessible experimentally due to the extremely fine length and time scales over which it occurs. In this paper, simulations of jerky flow in W-O interstitial solid solutions reveal three dynamic regimes emerging from the simulated strain rate-temperature space: one resembling standard solid solution strengthening, another one mimicking solute cloud formation, and a third one where dislocation/solute coevolution leads to jerky flow as a precursor of dynamic strain aging. The simulations are carried out in a stochastic framework that naturally captures rare events in a rigorous manner, providing atomistic resolution over diffusive time scales using no adjustable parameters.

## Abstract (web summary)

Understanding the plastic deformation mechanism within specific ranges of temperature and strain in metal alloys is of great technological importance. Here the authors report on dynamic simulations of dislocation–solute coevolution in tungsten crystals containing a few atomic parts per million of interstitial oxygen and their relation to unstable plastic flow.

## Introduction

**Fig. 1:**
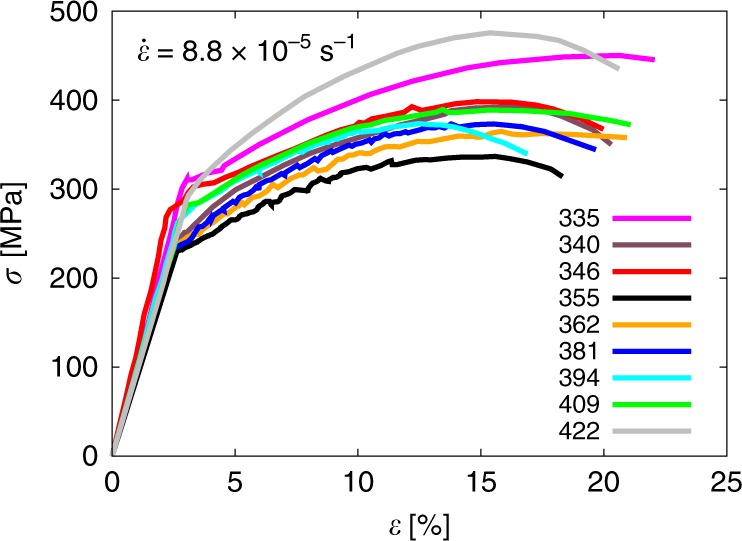
Serrated flow occurs in engineering alloys in specific temperature-strain rate windows. Deformation curves for Nb-0.75% at. O at fixed strain rate varying the temperature, displaying transitions from-smooth to serrated flow (adapted from ref. ^15^).

The Portevin-Le Chatelier (PLC) effect is a well-known phenomenon in materials science by which metallic alloys deform in an unstable manner, potentially leading to poor ductility and premature failure^1–18^. The PLC effect is characterized by jerky flow and the onset of inhomogeneous deformation, generally attributed to the dynamic interplay between dislocations and solute atoms. The macroscopic manifestation of this process is the appearance of serrated flow in the stress–strain (*σ*–*ε*) curve, a necessary—but not sufficient—condition to indicate the existence of dynamic strain aging (DSA). Figure 1 shows *σ*–*ε* curves of Nb-0.75O bulk crystals at different temperatures for a fixed strain rate. The figure exemplifies how changes in these two parameters can induce drastic variations in the plastic behavior of a material. Although there are other manifestations of DSA, the most important one is the inversion of the dependence of the strength, *σ*, on strain rate, $\dot \varepsilon$, resulting in a negative strain rate sensitivity (SRS, defined by an exponent, $m = {\mathrm{\Delta }}\sigma /{\mathrm{\Delta }}\log \dot \varepsilon$)^19^. The PLC effect belongs to a more general class of unstable phenomena in physics known as intermittent processes. These processes operate in a time-discontinuous manner and are pervasive in the natural world, being found across numerous scientific disciplines^20–22^. Modeling these processes is challenging both because their intrinsic dynamics are controlled by discrete events (i.e., fluctuations) and because the time and length scales governing fluctuations and the observed macroscopic response are often separated by many orders of magnitude.

**Fig. 2:**
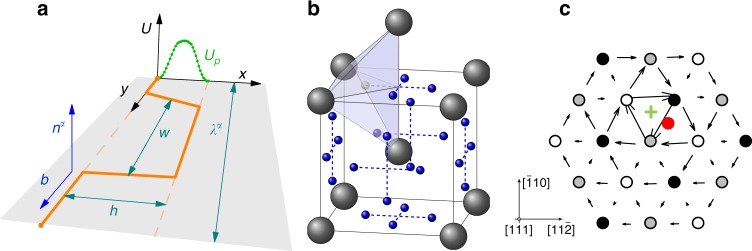
Crystallographic features of the oxygen-dislocation system. **a** Schematic diagram of the basic kink-pair geometry on the Peierls energy substrate *U*_p_(*x*). *b* and *n*^*α*^ are the Burgers vector and the normal of the glide plane *α*. $\lambda^{\alpha}$ is the length of an arbitrary straight screw dislocation segment on the same plane, and *h* and *w* are the periodicity of the substrate potential and the stable kink-pair separation, respectively (adapted with permission from ref. ^23^). **b** Elementary bcc lattice cell showing lattice atoms (in gray) and tetrahedral interstitial sites (in blue), including a shaded tetrahedron with the interstitial site highlighted in its center. **c** Differential displacement map obtained using DFT calculations showing the stable configuration for the dislocation core-O complex (oxygen atom shown in red, core position shown in green). The resulting core structure changes from so-called “easy” (no oxygen) to “hard” (with oxygen). Figure 2b, c adapted with permission from ref. ^39^.

Body-centered cubic (bcc) crystals are the basis of many technologically important metals and alloys. At low-to-moderate homologous temperatures, plastic slip in bcc materials is governed by the motion of screw dislocations on close-packed planes. Generally, this motion is understood to occur over a periodic energy landscape known as the Peierls potential *U*_P_ with a periodicity *h* and an amplitude *U*_0_. At low stresses, slip proceeds via the thermally activated nucleation of steps of height *h* on the dislocation line, known as kink pairs, and their subsequent sideward relaxation. The basic kink-pair geometry and the structure of the potential energy substrate are schematically depicted in Fig. 2a. The occurrence of these kinks makes dislocations in bcc metals behave as a many-body system, increasing the complexity of their treatment compared with other materials. Over five decades of research in bcc materials have conclusively revealed a direct connection between the kink-pair activation enthalpy and the temperature dependence of the flow stress in all pure metals^23^. In alloys, solutes are known to alter kink-pair nucleation and propagation rates giving rise to several well-known phenomena in bcc plasticity. For example, some substitutional bcc solid solutions are known to suffer a transition from solute softening to solute hardening as a function of alloy concentration^24–27^. This is now known to be a direct consequence of the interaction between solutes and kink-pairs^28^. Also, substitutional solutes are known to lower the so-called knee temperature, after which screw dislocation motion becomes athermal and the mobility of screw and non-screw segments becomes comparable^29^. Owing to their low diffusion rates and the absence of a vacancy-generation mechanism during plastic flow in dilute bcc alloys, dynamic strain aging is seldom attributed to substitutional solutes^15,16,30–32^, except perhaps at high temperatures or stresses. In the case of interstitial solid solutions, solute diffusion is effectively athermal, in the sense that in principle it is not the rate-limiting process, and—consequently—kink-pair nucleation dictates the duration of the waiting time in between plastic events. Under such conditions, dislocation motion proceeds in a discontinuous manner, with rapid slip bursts punctuated by localized trapping of dislocation cores by solute clouds.

Clearly, in view of the processes involved, the study of the microscopic mechanisms of DSA requires atomic resolution. However, intrinsic bottlenecks of atomistic simulations preclude their use for bridging the necessary time and length scale gap. In addition, the existence of kinks on screw dislocation segments in bcc crystals breaks the translational symmetry along the dislocation line, necessitating full three-dimensional models to capture the wavelengths of kink pairs along the line. This limits the applicability of techniques such as molecular dynamics (MD) to the mobility extremes of (i) stationary dislocations and highly mobile solutes (macroscopically equivalent to zero stress, high temperature conditions) and (ii), vice versa, mobile dislocations in a “frozen” solute field (high stress, low temperature). Bcc solid solutions pose yet extra challenges, as both governing mechanisms, i.e., solute diffusion and kink-pair nucleation, are considered rare events (albeit operating a priori on different timescales). Discrete event simulations, on the other hand, offer the possibility of handling rare events over a broad time scale spectrum within a relatively simple framework^33,34^. In this paper, we show for the first-time dynamic simulations of dislocation–solute coevolution in tungsten crystals containing trace amounts of interstitial oxygen (W-0.2 at.% O alloys), consistent with nominally pure metals contaminated through exposure to the environment^35,36^. Our simulations confirm that DSA occurs in a specific temperature-strain rate window that equalizes the timescales of solute transport and dislocation motion, resulting in intermittent slip and ultimately leading to negative SRS. This kind of simulations has been made possible by the recent development of a Kinetic Monte Carlo (KMC) model that accounts for thermally activated kink-pair nucleation and solute diffusion via stress field coupling and short-range inelastic interactions^37,38^. Our model is parameterized entirely using atomic scale calculations, as described in previous works^39^ (see Methods below) and is significantly more efficient than direct atomistic simulations. This allows us to study the relevant parameter space of stress, temperature, solute content, and dislocation line length to identify the conditions under which coevolution occurs.

## Results

### Energetics of oxygen atoms in W crystals containing a screw dislocation

In bcc W, oxygen atoms diffuse with a migration energy of 0.2 eV on a tetrahedral sublattice (shown in Fig. 2b)^39^. This makes the W-O system peculiar among other interstitial bcc solid solutions, where generally octahedral diffusion is favored^40,41^. When dislocations are present, oxygen diffusion suffers a drift owing to the underlying stress fields. In standard diffusion theory, this drift is characterized by an activation volume that—when coupled to the stress—yields the mechanical work to subtract from the activation energy for migration. In our case, the activation volumes of importance are approximately no larger than ¼ of one atomic volume, implying that O migration minimally perturbs neighboring W atoms when diffusing throughout the lattice. However, the trigonal distortions caused by oxygen atoms in their tetrahedral positions are most effectively neutralized near the screw dislocation core (see Fig. 2c), resulting in very stable bound structures with interaction energies of 1.2~1.8 eV^39,42^. As this is on the order of the kink-pair activation enthalpy (≈1.6 eV), oxygen-dislocation dissociation events become the rate-limiting step when solute diffusion is sufficiently fast (and/or dislocation motion sufficiently slow) to allow for the formation of solute clouds (akin to the so-called Cottrell atmospheres) around dislocation cores. Depending on whether this is or not the case, three scenarios may be considered based on the nature of dislocation–solute interactions:

(i) A low stress and high temperature regime where solute diffusion is favored over dislocation motion. One would then expect to see solute segregation at the dislocation core, i.e., solute atoms decorating the dislocation line with little or no dislocation glide. This is a widely studied scenario, both analytically and using atomistic methods^43–47^, corresponding to an adiabatic process where dislocation motion occurs over timescales much longer than solute transport.
(ii) A high stress and low temperature regime where dislocation glide dominates over solute motion. In this case, the alloy behaves in the manner of a substitutional solid solution, with stationary solute atoms interacting with moving dislocations. This is expected to lead to conventional solute hardening, which has also been studied extensively in the literature for bcc alloys by a number of different techniques^38,48–53^.
(iii) An intermediate stress and temperature region where solutes and dislocations display similar mobilities and evolve on comparable timescales. This is the region where DSA can occur^31,32^. However, such scenario has been comparatively much less studied using simulation methods (and even less so in bcc systems) owing to the intrinsic difficulties of treating two coevolving many-body systems. At present, this can only be studied via numerical simulation^54^ and is the primary subject of study in this work. In the following, we refer to this regime as the “coevolution regime”.

Here, “stress” and “temperature” are used to refer more or less generically to the mechanical and thermal driving forces. As discussed in Supplementary Note 1, the stress can be applied either directly (in a “stress-controlled” simulation) or indirectly via the application of a prescribed strain rate. The mode of application, however, does not affect the general description of the dynamic regimes (i), (ii), and (iii).

### KMC simulations of dislocation–solute coevolution

To mimic experimental tests, which are performed under constant strain rate, $\dot \varepsilon _0$, and temperature, *T*, conditions, we carry out strain rate-controlled simulations in wide range of $\dot \varepsilon _0$ and *T*. The instantaneous shear stress that results from a given prescribed (shear) strain rate is obtained as:

1 $$\tau (t) = 2\mu \left( {t\dot \varepsilon _0 - \varepsilon _p(t)} \right)$$

**Fig. 3:**
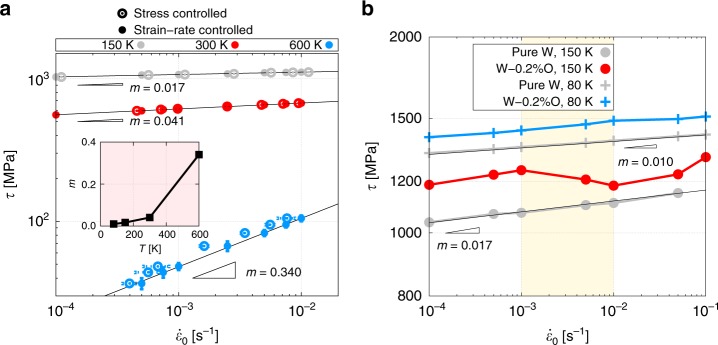
Negative strain rate sensitivity appears in alloys in specific strain rate-temperature range. **a** Comparison of the dependence of stress with strain rate (stress-controlled simulations) and vice versa (strain rate-controlled simulations) for a screw dislocation of length 400b in pure W at 150, 300, and 600 K. The results from both approaches are virtually identical. The strain rate sensitivity exponent, m, is displayed in the inset (as the slope in log-log space of each one of the *σ*-${\dot{\upvarepsilon}}$ curves). Error bars for the 150 and 300-K cases are smaller than the size of the markers used in the figure. **b** τ-${\dot{\upvarepsilon}}_0$ curves for the W-0.2%O system at 80 K (no solute motion) and 150 K (dislocation–solute coevolution). Although the alloy system shows the standard behavior at 80 K, inverse SRS can be appreciated between 10^–3^ and 10^–2^ s^–1^ at 150 K (shaded region). The pure W curves at 80 and 150 K are added for comparison (with the associated SRS exponents), revealing the hardening owing to the solute.

Where *t* is the total simulation time, *μ* is the shear modulus, and *ε*_*p*_ is the total accumulated plastic strain (calculated from the area on the glide plane swept by the dislocation as it moves, see Supplementary Note 1 for details). This stress is the response function in strain rate-controlled simulations, in contrast to stress-controlled simulations where the response function is the dislocation velocity. In pure W, for equal assumed dislocation densities, (this value of *ρ*_*d*_ also sets the dislocation line length to a magnitude of approximately $\lambda = \left( {\rho _d} \right)^{ - 1/2}$, which for *ρ*_*d*_ ≈ 1.4 × 10^14^ m^–2^ ^24^^,^ gives *λ* = 400*b*), both approaches yield identical results, as shown in Fig. 3a. The figure shows the dependence of the stress with the prescribed strain rate at 150, 300, and 600 K, characterized by SRS exponents between 0.34 and 0.02. However, as mentioned earlier, the most reliable marker for the onset of serrated flow in dilute alloys is in fact the observation of *m* *<* 0. To examine whether such a regime can be captured in our simulations, we analyze the behavior of the dislocation and the solute in W-0.2 at.% O in the 10^–4^ < $\dot \varepsilon _0$ < 10^–1^-s^–1^ and 80 < *T* < 300-K strain rate and temperature ranges. Figure 3b shows τ-$\dot \varepsilon _0$ curves at 80 and 150 K, clearly showing inverse SRS at 150 K between 10^–3^ and 10^–2^ s^–1^.

**Fig. 4:**
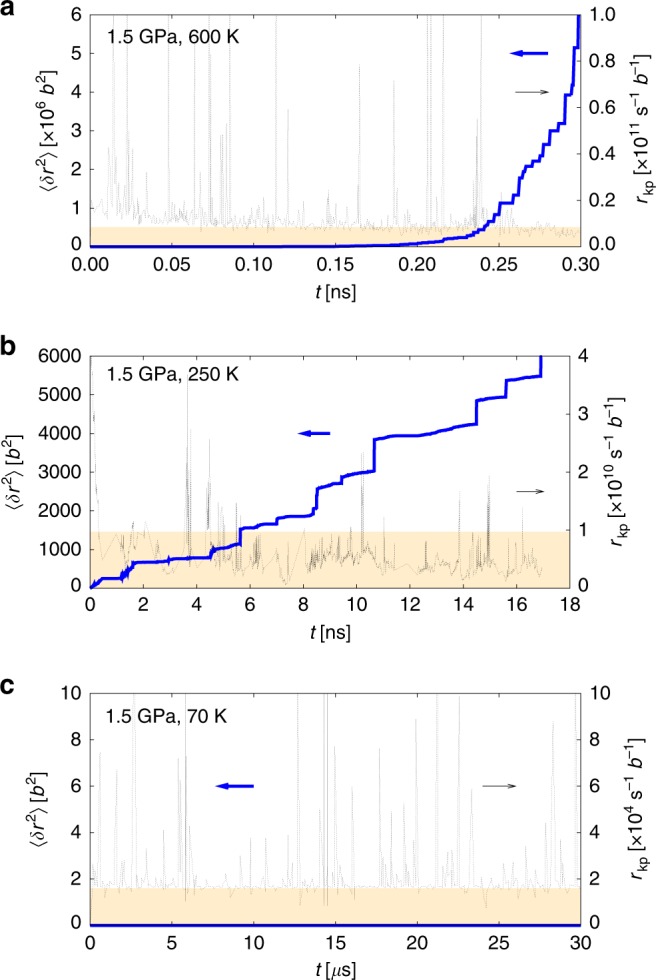
Solute migration is coupled to dislocation behavior. Evolution of the solute mean square displacement and kink-pair nucleation rates with time. The shaded band represents the equivalent kink-pair nucleation rate in pure W at each ${\dot{\upvarepsilon}}_0$-T condition. At a strain rate of 10^–4^ s^–1^ and a temperature of 150 K **a**, the dislocation and the solute appear to move in an uncorrelated fashion, with the dislocation experiencing kink-pair nucleation rates similar to the reference pure state and the solute following a non-linear mean square displacement. The sharp spikes in the kink-pair nucleation rate are manifestations of local variability in the solute spatial distribution and/or screw dislocation line configuration. At 5 × 10^–3^ s^–1^ and 150 K **b**, the solute is seen to undergo discontinuous motion, as revealed by a step-like mean square displacement, with the dislocation moving at a lower rate than in the homogeneous case and more kink-pair nucleation activity during stationary solute periods. At 5 × 10^–3^ s^–1^ and 80 K **c**, the solute becomes immobile and the dislocation moves through a static solid solution, with the kink-pair nucleation activity essentially following the homogeneous value with local spikes arising from dislocation–solute interactions. Simulation videos corresponding to these three cases can be seen in Supplementary Animations 1, 2, and 3.

Although this is in principle indicative of the onset of DSA on a “macroscopic” level, it is also important to study its microscopic manifestation, i.e., jerky (intermittent) plastic flow. To ascertain that this indeed corresponds to jerky flow, next we analyze the behavior of the solute by calculating its mean square displacement, *δr*^2^, as a function of time, whereas that of the dislocation is evaluated by tracking the evolution of the kink-pair nucleation rate, *r*_kp_. Figure 4 shows results for three characteristic strain rate-temperature points. The figure includes the expected kink-pair nucleation rate in a pure W crystal, represented for each $\dot \varepsilon _0$-*T* point as a background shaded band in each graph. Spikes in *r*_kp_ are manifestations of local compositional and/or configurational variabilities along the dislocation line in time. At 5.0 × 10^–5^ s^–1^ and 500 K (Fig. 4a), the dislocation and the solute appear to move in an uncorrelated fashion, with the dislocation experiencing kink-pair nucleation rates similar to the reference pure state and the solute following a non-linear mean square displacement. In fact, *δr*^2^ initially follows a parabolic evolution, which is indicative of the solute undergoing a biased random walk caused by the existence of a diffusion drift. At 5 × 10^–3^ s^–1^ and 150 K (Fig. 4b), the solute is seen to undergo discontinuous motion, as revealed by a step-like mean square displacement, with the dislocation moving at a lower rate than in the homogeneous case and more kink-pair nucleation activity during stationary solute periods. Finally, at 5 × 10^–3^ s^–1^ and 80 K (Fig. 4c), the solute becomes immobile and the dislocation moves through a static solid solution, with the kink-pair nucleation activity practically mimicking the homogeneous value with local spikes arising from dislocation–solute interactions. Simulation videos corresponding to these three cases can be seen in the Supplementary Information.

Although in Fig. 4a, c, the average kink-pair nucleation rate *r*_kp_ generally agrees well with the background pure W value, in 4b *r*_kp_ displays periods during which it is consistently lower than the reference. As well, the frequency of the local spikes is decreased with respect to the other two temperature cases. Correspondingly, the solute is seen to undergo an intermittent migration pattern, with periods of time where it is mostly stationary, and others characterized by rapid motion. We interpret this behavior as the precursor of dynamic strain ageing, where periods of reduced kink-pair nucleation activity are followed by stages of enhanced nucleation, leading to intermittent flow (whose macroscopic analog is serrated flow—as in Fig. 1).

**Fig. 5:**
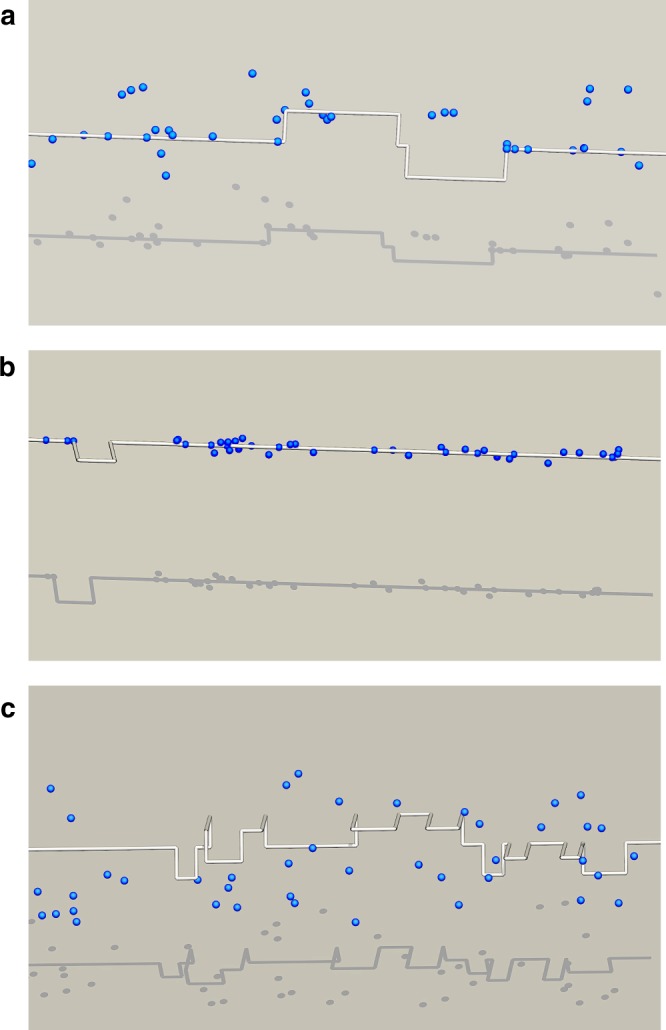
Geometric distribution of solute around dislocation lines. Simulation snapshots corresponding to the three dynamic scenarios described in the text. **a** Solute diffusion occurs over timescales much shorter than kink-pair nucleation, making both processes effectively decoupled. **b** The oxygen atoms segregate at the dislocation core, temporarily trapping it before the dislocation can release itself and produce a strain burst. **c** No solute motion is observed, and the dislocation moves in a static interstitial solid solution, developing more kink pairs and cross-kinks. The length of line captured in all three panels is approximately 100*b*. As it can be appreciated, the density and width of the kink pairs shown in the figures is markedly different from case to case.

Figure 5 shows high-resolution images of the dislocation–solute system, showing only dislocation segments and oxygen atoms. The images correspond to snapshots of 100*b*-long sections of the dislocation line for three scenarios similar to those shown in Fig. 4a–c. Kink pairs can be appreciated along the dislocation line in all three cases, with marked differences in density and kink-pair width clearly observed among them. In the coevolution regime (regime (iii)), the dislocation alternates episodes of solute pinning (as shown in Fig. 5b) with solute de-pinning, resulting in strain bursts marked by the formation of solute clouds around the dislocation core. Several kink pairs in Fig. 5c coexist on two different glide planes owing to the interaction between dislocation segments and static solutes, giving rise to the sporadic formation of cross-kinks^55^. A quantitative analysis of the solute density around the dislocation core for each of the three scenarios just described is also provided in Supplementary Note 2.

### Strain rate-inverse temperature diagrams and connection to DSA

**Fig. 6:**
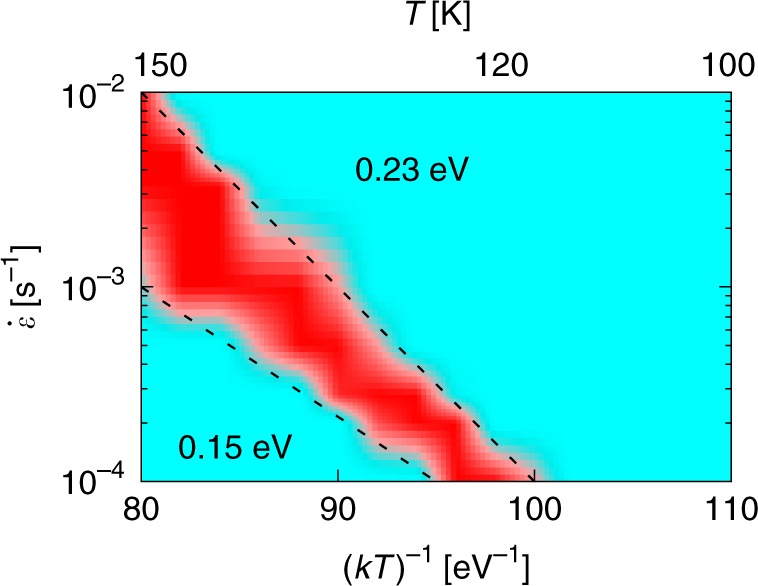
Using simulation results to map to engineering design curves. Color map of the strain rate-inverse temperature diagram obtained from our simulations. Each pixel in the figure is colored according to the propensity for jerky flow defined in the text, with red and cyan indicating high and low propensities, respectively. The dashed lines correspond to two different fits of the general equation $\dot \varepsilon \left( T \right) = \dot \varepsilon _0{\mathrm{exp}}\left( { - \frac{{\Delta E}}{{kT}}} \right)$, with the values of Δ*E* given next to them.

It is common in the literature to express the plastic behavior of dilute solid solutions displaying serrated flow as a strain rate-inverse temperature diagram highlighting the different regime transition boundaries. Figure 6 shows such a $\dot \varepsilon$-(*kT*)^–1^ chart, obtained by systematically examining all the strain rate-temperature combinations studied here. The figure shows the jerky flow region colored in red, delimited by two dashed lines that bound the limits within which discontinuous slip takes place. The slope of these lines defines two activation energies deduced from the expression $\dot \varepsilon \left( T \right) = \dot \varepsilon _0{\mathrm{exp}}\left( { - \frac{{{\mathrm{\Delta }}E}}{{kT}}} \right)$, marked in the figure as 0.15 and 0.23 eV, that characterize the operating mechanisms on each side of the jerky flow region. Given that the two principal processes governing the dislocation–solute system considered here are solute diffusion (with migration energies ranging between 0.15 and 0.20 eV) and kink-pair nucleation energies (ranging from 0 to 1.65 eV, see the Methods Section), the values shown in the graph can give an indication of the relative weight of each one on the overall dynamics. On the left side of the discontinuous flow region, the temperatures are sufficiently high to favor solute diffusion as the governing mechanism, with the value of 0.15 eV being consistent with this interpretation. On the right side, a higher energy of 0.23 eV suggests a more complex interplay between both processes, perhaps dominated by kink-pair nucleation next to oxygen atoms and more-limited solute diffusivity.

## Discussion

Although our simulations pertain to one single dislocation source, we believe that they are still representative of bulk material behavior—which is the context in which DSA is strictly defined—capturing directly the interplay between mutually coevolving dislocations and solute atoms in a way that allow us to explain observed macroscopic behavior. As such, they offer a unique window into the governing mechanisms behind serrated flow and the PLC effect. It must also be kept in mind that bcc metals display a set of particularities that call for the development of very specific approaches. One of these is the reduction of crystal plasticity to the behavior of a single screw dislocation source under single slip conditions, as has been confirmed in a number of studies^23,28,31,32^. Thus, one can think of our model as descriptive of the characteristic behavior of dislocation sources in crystal grains of bcc materials, such that the total strain at the bulk level can be additively obtained from the different contributions of sources operating in the manner described here.

In quantitative terms, our results are highly influenced by the choice alloy made in this work. DSA in real W alloys may occur in a different regime than that shown in Fig. 6, a reflection of the existence of a much-less simplistic microstructure and chemical composition than the one considered here. In this sense, the solute concentrations, solute species, dislocation densities, and dislocation types used here all represent an idealized version of real alloys and their behavior. However, our intent in this paper is to study the physics behind the interplay between dislocations and solute atoms in interstitial alloys and unveil the fundamental mechanism governing an elusive phenomenon at the atomic scale as is DSA and the PLC effect.

In conclusion, we have shown using discrete stochastic models of dislocation–solute coevolution that “jerky” flow in dilute bcc interstitial solid solutions is a natural consequence of the dynamic interplay between the motion of solutes and dislocations. To capture such coevolution, the computational model must be capable of operating on “diffusive” timescales, i.e., above the characteristic atomic vibration period, on which thermally activated processes take place. Our model is able to explore such regimes, exposing strain rate-inverse temperature maps that define the dynamic behavior of the alloy. Although these simulations pertain to one dislocation only, we are confident that the results reported here can be helpful in interpreting the alloy behavior at the level of the microstructure. Then, it is at that level that dynamic strain ageing is defined, and where discontinuous flow can lead to localization and embrittlement.

## Methods

### DFT calculations

All density functional theory (DFT) calculations were carried out using the Vienna Ab Initio Simulation Package code^56^ with the projector augmented wave^57,58^ pseudopotential scheme within the Perdew-Burke-Ernzerhof-generalized gradient approximation. A 400-eV kinetic-energy cutoff was used, and the Hermite-Gaussian scheme was employed, with a smearing of 0.2 eV for electronic occupation. Dislocations were modeled by inserting a dislocation dipole in a 135-atom simulation cell using periodic boundary conditions, as usually done for bcc metals^42,59–62^. Oxygen atoms were inserted in tetrahedral positions up to the 6th nearest-neighbor to the dislocation core and the dislocation-oxygen system was relaxed until the forces on all the atoms were less than 2 × 10^−2^ eV/Å. Details of the calculations and additional results are provided in ref. ^39^.

### KMC simulations

The KMC method evolves a system through a sequence of states via a random walk process. All the transitions connecting a given state with the neighboring states are defined by their transition rate. These rates are sampled with the correct probability to simulate the time evolution of the system. In our case, we have three main classes of transitions: (i) kink-pair nucleation, (ii) kink propagation and/or de-trapping, and (iii) solute diffusion. Our approach is a three-dimensional, full-elasticity model of arbitrary screw dislocation geometries in bcc lattices that accounts for slip on all {110} planes of the [111] zone. To ensure detailed balance, each solute atom is assigned a complete set of possible transitions to all possible neighboring sites. Kink pairs along a given dislocation segment of length *λ* are nucleated with a probability per unit time:

2 $$r_{{\mathrm{kp}}}\left( {T,\tau } \right) = \nu^{\prime} \left( {\frac{{\lambda - w}}{b}} \right){\mathrm{exp}}\left( { - \frac{{\Delta H_{{\mathrm{kp}}}\left( \tau \right)}}{{kT}}} \right)$$

where *ν*′ is an attempt frequency, *w* is the kink-pair separation (see Figure 1a), and, $\Delta H_{{\mathrm{kp}}}$ is the activation enthalpy, which is a function of the local resolved shear stress τ. *k* is Boltzmann’s constant, *b* the modulus of the Burgers vector, and *T* the absolute temperature. The above rate is calculated for all segments with *λ* > *w* on all possible glide planes, where *w* is the kink-pair width, sampled for each value of *τ* from a function obtained from atomistic calculations (described in ref. ^37^). *τ* is calculated for each glide system from the stress tensor *σ* (Fig. 2a), which includes corrections for non-Schmid effects^37,38^.

The total stress at each spatial point ***r*** includes contributions from the applied strain rate or applied stress (as described in Supplementary Note 1), all dislocation segments, and solute atoms. In this work, *σ* is oriented as to make the main (110) glide plane be the maximum resolved shear stress plane (MRSSP). However, thermal activation, spatial stress fluctuations, as well as local interactions with solute atoms, can all contribute to non-MRSSP kink-pair nucleation and glide. In fact, that our model can naturally capture all this variability is a principal reason why we can tackle complex problems such as the present one. No intermittent flow can occur in our simulations from confined-volume plasticity^63–67^, and thus all instances of jerky flow can only be caused by dislocation–solute interactions.

For their part, oxygen atoms move throughout the tetrahedral sublattice according to the following jump rate:

3 $$\nu _{{\mathrm{Ox}}}\left( {T,{\it{\sigma }}\left( {\it{r}} \right)} \right) = \nu^{\prime\prime} {\mathrm{exp}}\left( { - \frac{{\Delta H_{{\mathrm{Ox}}}\left( {{\it{\sigma }}\left( {\it{r}} \right)} \right)}}{{kT}}} \right)$$

where *ν*″ is also an attempt frequency, $\Delta H_{{\mathrm{Ox}}}$ is the activation enthalpy, itself a function of the migration energy, *E*_*m*_, the heat of solution, ∆*H*_*s*_, and the mechanical energy *W*_*m*.:_ $\Delta H_{{\mathrm{Ox}}} = E_m + \Delta H_s - W_m$. All of these also depend linearly on the stress state *σ*, with proportionality constants given in ref. ^39^. Kink translation is assumed to occur athermally unless kinks interact with solute atoms, in which case kink de-trapping is also dealt with in a thermally activated fashion with binding energies between 1.5 and 1.8 eV^39^.

## Supplementary information

Supplementary Information

Description of Additional Supplementary Files

Supplementary Movie 1

Supplementary Movie 2

Supplementary Movie 3

## Data Availability

All computational and/or experimental data in CSV format can be provided upon request.

## Code availability

The KMC code is available in open source form at: http://jmarian.bol.ucla.edu/packages/packages.html (entry #5).
