# Supplementary material for: Simulating the mechanisms of serrated flow in interstitial alloys with atomic resolution over diffusive timescales
Source: Nat Commun. 2020 Mar 6;11:1227. doi: 10.1038/s41467-020-15085-3 (PMC7060222; doi:10.1038/s41467-020-15085-3)
Supplement: Supplementary file 1 — Supplementary Information [file 41467_2020_15085_MOESM1_ESM.pdf]

“Unveiling the mechanisms of serrated flow in dilute interstitial alloys using simulations with atomic resolution over diffusive timescales”

Zhao et al.

Supplementary Information

This document contains additional analysis in support of our conclusions in the main manuscript. First, we describe the implementation of strain rate-controlled simulations. This is followed by a quantitative study the solute-dislocation behavior behind the three animation files supplied as supplementary information.

Supplementary Note 1: Strain rate-controlled vs stress-controlled simulations

The governing (tensorial) expressions for strain rate-controlled simulations are:

$$\boldsymbol{\sigma}(t) = \mathbb{C}\left(t\dot{\boldsymbol{\varepsilon}}^0 - \boldsymbol{\varepsilon}^{\mathrm{p}}(t)\right) \tag{1}$$

$$\boldsymbol{\varepsilon}^{\mathrm{p}}(t^{n+1}) = \boldsymbol{\varepsilon}^{\mathrm{p}}(t^n) + \delta\boldsymbol{\varepsilon}^{\mathrm{p}}(t^{n+1}) \tag{2}$$

$$\delta\boldsymbol{\varepsilon}^{\mathrm{p}}(t) = \frac{\boldsymbol{s}\otimes\boldsymbol{n}+\boldsymbol{n}\otimes\boldsymbol{s}}{2}\left(\frac{b}{L}\right)\rho_d\delta A(t) \tag{3}$$

where $\mathbb{C}$ is the elasticity matrix, $\dot{\boldsymbol{\varepsilon}}^0$ is the prescribed strain rate tensor, $\boldsymbol{\varepsilon}^{\mathrm{p}}(t)$ is the accumulated plastic strain tensor, ρ_d is the dislocation density (constant), $\boldsymbol{n}$ and $\boldsymbol{s} = \boldsymbol{b}/b$ are the glide plane normal and the slip direction, respectively ($\boldsymbol{b}$ and b are the Burgers vector and its modulus), L is the dislocation line length, and δA is the area swept by the dislocation during a given δt. The term $\left(\frac{\boldsymbol{s}\otimes\boldsymbol{n}+\boldsymbol{n}\otimes\boldsymbol{s}}{2}\right)$ is known as the Schmid tensor and is used to obtain the appropriate projection of δA. As the only component of $\dot{\boldsymbol{\varepsilon}}^0$ that produces a nonzero resolved shear stress is a shear strain rate of the zy type (in accordance with the geometry described in ref. [38] of the main manuscript), the relevant applied stress component is obtained as:

$$\tau_{zy}(t) = 2\mu\left(t\dot{\varepsilon}_{zy}^0 - \varepsilon_{zy}^p(t)\right) \tag{4}$$

with

$$\varepsilon_{zy}^p(t^{n+1}) = \varepsilon_{zy}^p(t^n) + \delta\varepsilon_{zy}^p(t^{n+1}) \tag{5}$$

where μ is the shear modulus and δt^{n+1} is the current timestep. Recall that t is the total (accumulated) time. In the present setup, with $\boldsymbol{s} = [001]$ and $\boldsymbol{n} = \left[n_x\ n_y\ 0\right]$, the plastic strain update is simply obtained as:

$$\delta\varepsilon_{zy}^p = \frac{\rho_d n_y b\ \delta A}{2L} \tag{6}$$

where ρ_d is the dislocation density and b is the modulus of the Burgers vector. δA is calculated depending on whether the event is a kink pair nucleation event, or the propagation of a kink. In each case, the area swept at a given time t^{n+1} is written as:

$$\delta A = \begin{cases} hw(\tau_{\mathrm{RSS}}), \text{ if the event is a kink-pair nucleation} \\ h\ \delta t\ v_k(\tau_{\mathrm{RSS}}), \text{ if the event is kink propagation} \end{cases}$$

where h is the periodicity along the $\langle 112\rangle$ direction in the bcc lattice, w is the kink separation in a kink pair, and v_k is the kink velocity. Both w and v_k directly depend on the resolved shear stress at each local point. From eq. (4), the external contribution to τ_{RSS} is simply τ_{zy}. The area swept by a kink as it moves can also be obtained directly as $(h\ \delta x)$ to include all other contributions to

the kink motion such as when diffusive or solute processes are in operation (cf. Section 2.1 in ref [38] of the main manuscript). This is the case when a kink becomes trapped at a solute atom: it needs thermal assistance to overcome the trap and continue its motion. When this happens, the kink is displaced by a distance δx away from the solute which is then used to calculate δA. Time-dependent stress evolution curves corresponding to dislocation motion in pure W and to jerky W-0.2%O are shown in Figures 1a and 1b.

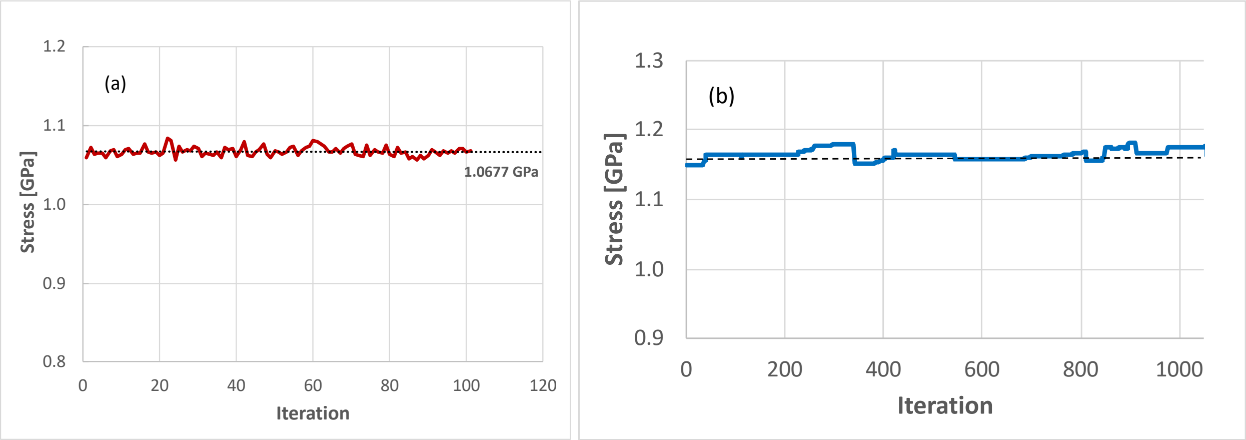

***Supplementary Figure 1**: **Differences between stress and strain rate-controlled simulations in W-O alloys**.*

(a) Evolution of the stress with time during a strain rate-controlled simulation of dislocation motion in pure W at 150 K and 10^{-3} s^{-1}. The stress oscillates around an average value of 1.067 GPa. (b) Evolution of the stress with time during a strain rate-controlled simulation of dislocation motion in W-0.2% at. O at 150 K and 10^{-3} s^{-1}. The stress suffers more abrupt changes due to the interactions between solutes and dislocations.

For its part, in stress-controlled simulations, the strain rate is obtained from the dislocation velocities calculated at each stress using Orowan's equation, as:

$$\dot{\varepsilon}(\tau) = \rho_d b v(\tau_{\mathrm{RSS}}) \tag{7}$$

where $v(\tau)$ is the dislocation velocity, which depends also on the resolved shear stress τ_{RSS}. In a pure system, both approaches –stress-controlled and strain-rate controlled– must yield equivalent results in steady state conditions, i.e. when the dislocation velocity reaches its constant value. Indeed, this is what is seen in Figure 2, where the strain rate calculated from the dislocation velocity and Orowan's equation (stress-control, eq (7)) in pure W is practically identical to the strain rate when prescribed directly (strain-rate control), eq. (1).

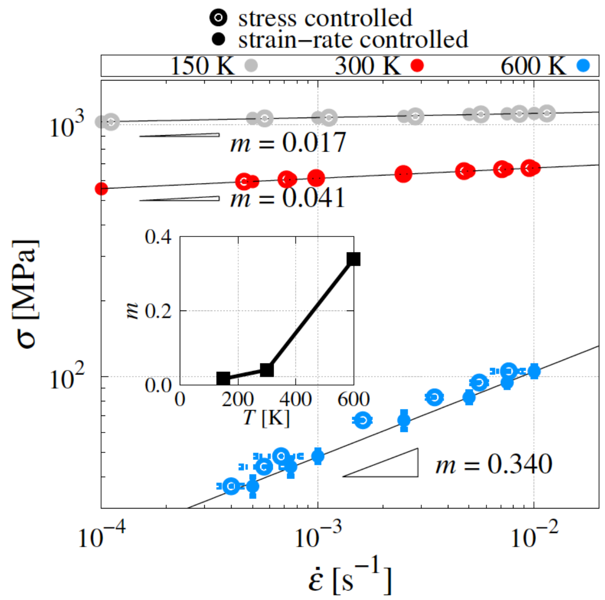

Supplementary Figure 2**: **Equivalence between stress and strain rate-controlled simulations in pure W.

Comparison of the dependence of stress with strain-rate (stress-controlled simulations) and vice versa (strain-rate controlled simulations) for a screw dislocation of length 400b in pure W at 150, 300, and 600 K. The results from both approaches are virtually identical. The strain-rate sensitivity exponent, m, is displayed in the inset (as the slope in log-log space of each one of the $\boldsymbol{\sigma}$-$\dot{\boldsymbol{\varepsilon}}$ curves). Error bars for the 150 and 300-K cases are smaller than the size of the markers used in the figure.

Supplementary Note 2: Dynamic solute distribution around the dislocation line

Here we track the solute density $\rho(r,t)$ as a function of distance to the dislocation core. To eliminate the time dependence and obtain a 'static' picture of the solute distribution for ease of analysis, we discretize the space around the core into a series of concentric cylindrical volumes increasing in thickness by an amount b. This is schematically depicted in Figure 3. We then time average the solute concentration within each cylindrical slice as:

$$c(r) = \frac{1}{t'}\int_0^{t'} \rho(r,t)dt \tag{8}$$

So that $c(r)$ is a 'still' picture of the solute radial distribution around the dislocation core averaged over a sufficiently long-time t'. It must be noted, however, that the position of the dislocation itself changes with time, and so r is referred to a local frame of reference with origin at the dislocation center of mass $\mathbf{COM}(t)$:

$$\boldsymbol{r} = \boldsymbol{r}' - \boldsymbol{COM}(t) \tag{9}$$

where $\boldsymbol{r}'$ is the location of a solute atoms referred to a global coordinate system, and bold script indicates vectorial quantities in three dimensions. Note that the function(s) $\mathbf{COM}(t)$ are a direct output of the kinetic Monte Carlo simulations. The above two equations are linked by $r = \|\boldsymbol{r}\|$. This analysis is done in a highly dynamic environment, with solute atoms moving in and out of each cylindrical slice either by direct diffusion or as the dislocation moves in space and r changes. Then, we count how many solute atoms occupy each slice at each time and then divide by the slice volume to obtain the solute density:

$$\rho(r,t) = \frac{n(r,t)}{V(r)} \quad (10)$$

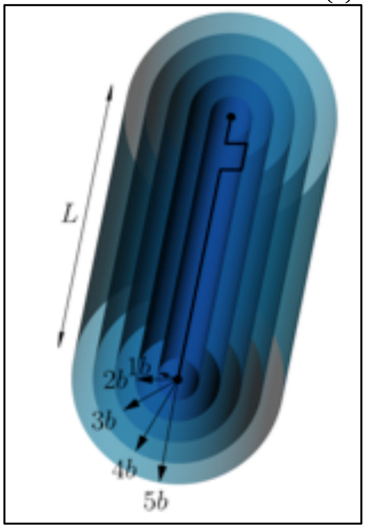

***Supplementary Figure 3: Geometry used to obtain the radial distribution of solute around dislocation line**.*
Schematic diagram showing the discretization of volume around the dislocation line into cylindrical slices to track the dynamic evolution of solute. The dislocation line length is L, and a kink-pair is shown for descriptive purposes.

However, for the discretization mentioned above, this density can be simplified as:

$$\rho(m,t) = \frac{n(m,t)}{\pi b^2 L(2m+1)} \quad (11)$$

Where L is the total dislocation length ($400b$ in this work) and m is the slice index of thickness b ($m = 1,\dots 6$, see Fig. 1). We then apply this analysis in a series of simulations at the same temperature of 150 K varying the strain rate to encompass the three regions of dynamic behavior depicted in Figure 4 of the main manuscript.

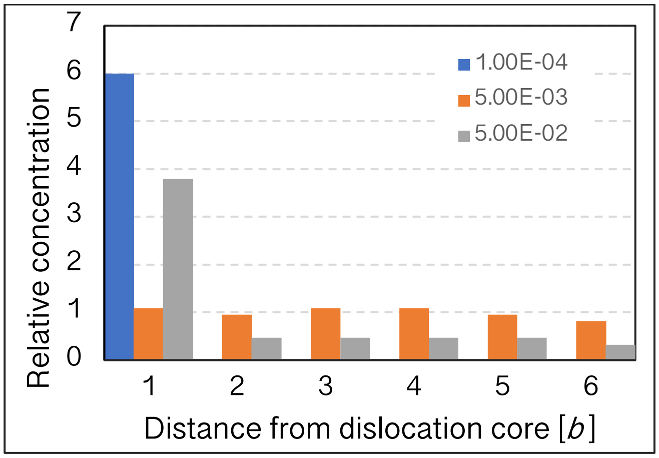

Supplementary Figure 4: Time-integrated solute distribution around the dislocation line.
Solute concentration histogram showing the average distribution of oxygen atoms around the dislocation core expressed relative to the alloy composition of 0.2% at. The data correspond to three different

simulations at 10^{-4}, $5\times10^{-3}$and 5×10^{-2} s^{-1} all at 150 K. These distributions are time-averaged over several ns of simulation. As such, the data represent spatially and time integrated results.

The results are shown in Figure 4. Solute concentrations are expressed relative to the nominal alloy concentration of 0.2% at., such that the background solute density is expressed as unity. The three cases shown are:

- 10^{-4} s^{-1} (blue), corresponding to the high solute mobility and little or no dislocation glide (refer to Fig. 3 of the main text). At this stress, the solute segregates on the dislocation line, resulting in a six-fold increase of the solute concentration at the core, and a complete depletion (like a 'denuded' zone) out to a distance of $6b$. This corresponds to animation '150K_Segr_400b_0.2%.mov' provided as part of this Supplementary Information. The far-field concentration remains constant at a value of 0.2% at.
- 5×10^{-2} s^{-1} (orange), in the region with high dislocation mobility with limited solute diffusion. Here, the dislocation moves too fast for the solute to respond to the dislocation stress fields. The result is a homogenous solute distribution and the dynamics resembles that of standard solid solution hardening. This corresponds to animation '150K_SSol_400b_0.2%.mov'.
- 5×10^{-3} s^{-1} (gray), found inside the region of jerky flow in Fig. 4 of the main manuscript, shows a local solute enrichment near the core, although smaller in magnitude than at 10^{-4} s^{-1} MPa, and no complete depletion. This is indicative of a 'cloud' of solute that chases and traps the dislocation in a time-discontinuous manner. That is, the graph shows a time-averaged distribution that results from a sequence of alternating (local) enrichment/depletion processes occurring in and intermittent way. This corresponds to animation '150K_Jerky_400b_0.2%.mov'.
