# Supplementary material for: Simulating the mechanisms of serrated flow in interstitial alloys with atomic resolution over diffusive timescales
Source: Nat Commun. 2020 Mar 6;11:1227. doi: 10.1038/s41467-020-15085-3 (PMC7060222; doi:10.1038/s41467-020-15085-3)
Supplement: Supplementary file 2 — Description of Additional Supplementary Files [file 41467_2020_15085_MOESM2_ESM.pdf]

Description of Additional Supplementary Files

File name: Supplementary Movie 1
Description: Simulation at a strain rate of 10–4 s–1 and 150 K showing solute segregation on the dislocation line. This is akin to the formation of pinning atmospheres of rapidly-moving solutes on slowly-gliding dislocations.

File name: Supplementary Movie 2
Description: Simulation at a strain rate of 5×10–2 s–1 and 150 K showing a screw dislocation moving through a field of static solute atoms.

File name: Supplementary Movie 3
Description: Simulation at a strain rate of 5×10–3 s–1 and 150 K showing dislocation solute coevolution. This is representative of inverse strain rate sensitivity conditions and dynamic strain aging.
